# Patients with pheochromocytoma exhibit low aldosterone renin ratio-preliminary reports

**DOI:** 10.1186/s12902-020-00620-6

**Published:** 2020-09-11

**Authors:** Tomoko Yamada, Hidenori Fukuoka, Yusei Hosokawa, Yukiko Odake, Kenichi Yoshida, Ryusaku Matsumoto, Hironori Bando, Yuko Okada, Yushi Hirota, Genzo Iguchi, Wataru Ogawa, Yutaka Takahashi

**Affiliations:** 1 Division of Diabetes and Endocrinology, Kobe University Hospital, 7-5-2 Kusunoki-cho, Chuo-ku, Kobe, 650-0017 Japan; 2 Division of Diabetes and Endocrinology, Kobe University Graduate School of Medicine, 7-5-2 Kusunoki-cho, Chuo-ku, Kobe, 650-0017 Japan

**Keywords:** Pheochromocytoma, Adrenal incidentaloma, Renin, Aldosterone, ARR

## Abstract

**Background:**

Plasma renin activity (PRA) is generally increased in patients with pheochromocytoma (PCC) due to low circulating plasma volume and activation of β-1 adrenergic receptor signaling. However, there has been no study on the aldosterone renin ratio (ARR) in patients with PCC. To elucidate the issue, this study aimed to determine the PRA, plasma aldosterone concentration (PAC), and ARR in patients with PCC and compare them with those in patients with subclinical Cushing’s syndrome (SCS) and non-functioning adrenal adenoma (NFA).

**Methods:**

In this retrospective single-center, cross-sectional study, 67 consecutive patients with adrenal tumors (PCC (*n* = 18), SCS (*n* = 18), and NFA (*n* = 31)) diagnosed at Kobe University Hospital between 2008 and 2014 were enrolled.

**Results:**

PRA was significantly higher in patients with PCC than in those with SCS and NFA (2.1 (1.3 ~ 2.8) vs. 0.7 (0.5 ~ 1.8) and 0.9 (0.6 ~ 1.4) ng/mL/h; *p* = 0.018 and *p* = 0.025). Although PACs were comparable among the three groups, ARR was significantly lower in patients with PCC than in those with SCS and NFA (70.5 (45.5 ~ 79.5) vs. 156.0 (92.9 ~ 194.5) and 114.9 (90.1 ~ 153.4); *p* = 0.001 and *p* < 0.001). Receiver operating characteristic curve analysis demonstrated that, in differentiating PCC from NFA, PRA > 1.55 ng/mL/h showed a sensitivity of 70.0% and specificity of 80.6%. Interestingly, ARR < 95.4 showed a sensitivity of 83.3% and specificity of 86.7%, which were higher than those in PRA.

**Conclusions:**

ARR decreased in patients with PCC, which was a more sensitive marker than PRA. Further study is necessary to understand the usefulness of this convenient marker in the detection of PCC.

**Trial registration:**

This study was not registered because of the retrospective analysis.

## Background

Pheochromocytoma (PCC) is a tumor arising from adrenomedullary chromaffin cells that commonly secret catecholamines (CAs), namely, adrenaline (Ad), noradrenaline (NA), and dopamine (DA). CAs can stimulate all major adrenergic receptors, including α1, α2, β1, and β2 receptors. Activation of α1 adrenergic receptors, located in vascular walls, induces significant vasoconstriction, which chronically causes hypovolemia. Patients with PCC require surgical treatment to prevent fatal changes in hemodynamics [1]. Since life-threating problems, such as hypovolemic shock, can occur during the perioperative period, α1 blocker with salt loading should be administered to patients with PCC before surgery. These preoperative treatments can correct vasoconstriction and improve extracellular fluid volume [2]. Therefore, preoperative diagnosis and/or exclusion of PCC is quite important.

Measurement of plasma or urinary metanephrine (uMN) and normetanephrine (uNMN) levels is the gold standard in PCC screening. Because of its high sensitivity and specificity, it is quite useful in detecting and excluding pheochromocytoma [3–7]. However, a part of patients with PCC exhibit normal levels or within two-fold of the upper limit of normal levels [8]. Additionally, false-positive results for uMN or uNMN make it difficult to exclude PCC. Furthermore, in general practice, simple screening markers are needed to detect PCC from patients with hypertension.

Plasma renin activity (PRA) is increased in patients with PCC due to low circulating plasma volume and activation of β-1 adrenergic receptor signaling [9, 10]. In the differential diagnosis of adrenal incidentaloma (AI) or secondary hypertension, plasma aldosterone concentration (PAC) and PRA are routinely measured in the screening of primary aldosteronism. However, limited data have been reported regarding PAC, PRA, and aldosterone renin ratio (ARR) in patients with PCC. This study aimed to determine the difference in PAC, PRA, and ARR between patients with PCC and those with subclinical Cushing’s syndrome (SCS) or non-functioning adrenal adenoma (NFA).

## Methods

### Subjects and study design

This retrospective cross-sectional single-center study was approved by the ethics committee of Kobe University Hospital, and written informed consent was obtained from all subjects (IRB #1351). We enrolled 83 consecutive patients with PCC, SCS, and NFA who were diagnosed and hospitalized in Kobe University Hospital between 2008 and 2014. We confirmed that all of these subjects had both PRA and PAC evaluated. Among them, patients who had received medication that can affect the renin-angiotensin aldosterone system, including aldosterone receptor blockers, diuretics, angiotensin converting enzyme (ACE) inhibitors, angiotensin receptor blockers (ARB), and β-adrenergic blockers (*n* = 16), were excluded. The diagnosis of PCC was based on the Endocrine Society Guideline [11], which focused on increased uMN or uNMN levels, tumor demonstration by imaging test, and positive ^123^I- metaiodobenzylguanidine (MIBG) scintigraphy. In all cases of PCC, the final diagnosis was histologically confirmed after surgery. The diagnosis of SCS and NFA was performed based on each guideline [12, 13].

### Hormone assays

The PRA, PAC, and plasma CA levels were measured in the morning after overnight fasting in the supine position. After 24-h urinary excretion of fractionated MNs and CAs, all subjects were instructed to abstain from caffeinated foods and drinks for at least 48 h. PRA, PAC, and plasma CA levels were measured by high-performance liquid chromatography (HPLC) (LSI Medience Corporation, Tokyo, Japan), enzyme immunoassay (BML, Inc., Tokyo, Japan), and radioimmunoassay (LSI Medience Corporation, Tokyo, Japan), respectively. The intra- and inter assay coefficients of variations for each hormone assay were as follows: PRA, < 10 and < 15%; PAC, < 7.8 and < 10.6%; plasma Ad, < 4.08 and < 2.23%; NA, < 9.34 and < 2.27%; and DA, < 8.96 and < 2.89%. uMN and CA levels were measured by HPLC (LSI Medience Corporation, Tokyo, Japan). The intra- and inter assay coefficients of variations for each hormone assay were as follows: urinary Ad, < 6.21 and < 6.35%; urinary NA, < 4.09 and < 3.82%; urinary DA, < 5.32 and < 4.46%; uMN, < 1.4 and < 6.2%; and uNMN, < 0.7 and < 5.3%.

### Statistical analysis

Data were appropriately expressed as mean ± standard deviation or median (interquartile range). These data were logarithmically transformed to normality before statistical analysis. We defined PCC as a control, and compared to SCS and NFA. Statistical comparisons among patients with PCC, SCS, and NFA were made using the Kruskal-Wallis test with post hoc Bonferroni’s test or χ^2^ test followed by Tukey’s honestly significant difference test, as appropriate. Receiver operating characteristic (ROC) curve analysis was performed to determine the optimal cutoff value in the diagnosis of PCC. *P*-values < 0.05 were considered statistically significant. Statistical analyses were performed using with SPSS version 22.0 for Windows (SPSS Inc., Chicago, IL).

## Results

### Patient characteristics

As a result, we analyzed a total of 67 patients, consisting of 18 patients diagnosed with PCC, 18 patients with SCS, and 31 patients with NFA. The clinical characteristics of 67 patients (PCC; *n* = 18, SCS; *n* = 18, and NFA; *n* = 31) are shown in Table 1. Patients consist of 28 men and 39 women (women, 58.2%), the mean age was 57 ± 15 years, and the mean body mass index (BMI) was 23.1 ± 4.1 kg/m^2^. The mean size of the tumor was 3.3 ± 2.6 cm. Twenty-seven patients (40.3%) had hypertension and treated with calcium channel blocker (CCB) and/or α-blocker. Among the three groups, BMI in the PCC group was significantly lower than that in the SCS group (21.0 ± 3.3 vs. 24.9 ± 4.7 kg/m^2^, *p* = 0.019). One patient with PCC was diagnosed with a familial syndrome, von Hippel-Lindau disease.

**Table 1:** Clinical characteristics of patients with PCC, SCS, and NFA

	Total	PCC	SCS	NFA	*p value*		
					*Kruskal-Wallis*	PCC vs SCS	PCC vs NFA
Number	67	18	18	31			
Age (yr)	57 ± 15	53 ± 19	57 ± 14	59 ± 14	0.404	–	–
Sex (males/females)	28 / 39	7 / 11	6 / 12	15 / 16	0.564	–	–
Body Mass Index (kg/m^2^)	23.1 ± 4.1	21.0 ± 3.3*	24.9 ± 4.7*	23.3 ± 3.7	0.023	0.019	0.256
Systolic blood pressure (mmHg)	127 ± 19	126 ± 20	132 ± 12	126 ± 22	0.372	–	–
Diastolic blood pressure (mmHg)	74 ± 11	72 ± 9	77 ± 11	73 ± 12	0.370	–	–
Antihypertensive Drugs (%)							
CCB	25 (37%)	5 (28%)	8 (44%)	12 (39%)	0.572	–	–
α blocker	7 (10%)	3 (17%)	2 (11%)	2(6%)	0.527	–	–

*CCB* Calcium channel blocker, *PCC* pheochromocytoma, *SCS* subclinical cushing syndrome, *NFA* Non-functioning adrenal tumor

Data are expressed as mean ± S.D. *p <0.05 compared between group of PCC and SCS

### Endocrinological findings

Measurements of CA level and its metabolites in plasma and urine, adrenocorticotropin (ACTH), cortisol (F) in the morning, and F after 1-mg dexamethasone suppression test (DST), and dehydroepiandrosterone sulfate (DHEA-S), and imaging findings including tumor size and CT value are shown in Table 2. As expected, levels of CA and its metabolites in urine in patients with PCC were significantly higher than those in patients with SCS and NFA. Plasma ACTH level was lower in patients with SCS than those with PCC (9.7 (8.9 ~ 14.1) vs. 29.8 (22.8 ~ 40.3) pg/mL, *p* < 0.001), and serum F level after 1-mg DST was higher in patients with SCS than that in patients with PCC (6.1 (4.0 ~ 10.6) vs. 1.5 (1.2 ~ 1.7) μg/dL, *p* < 0.001).

**Table 2:** Biochemical values and tumor characteristics in patients with PCC, SCS and NFA

	PCC	SCS	NFA	*p value*		
				*Kruskal-Wallis*	PCC vs SCS	PCC vs NFA
**Plasma Ad (pmol/mL)**	0.13^¶^ (0.05–0.29)	0.03 (0.02–0.03)	0.03^¶^ (0.02–0.04)	0.018	0.080	0.044
**NA (pmol/mL)**	0.47^*¶^ (0.28–3.10)	0.25^*^ (0.16–0.35)	0.28^¶^ (0.16–0.40)	0.008	0.027	0.014
**Urine Ad (μmol/day)**	38.8^**¶^ (10.6–98.6)	6.13^**^ (4.63–7.48)	7.2^¶^ (4.35–8.88)	< 0.001	< 0.001	< 0.001
**NA (μmol/day)**	356^**¶¶^ (166–1004)	99^**^ (85–114)	110^¶¶^ (87–149)	< 0.001	< 0.001	< 0.001
**MN (μmol/day)**	0.74^**¶¶^ (0.13–2.23)	0.09^**^ (0.08–0.09)	0.10^¶¶^ (0.07–0.11)	< 0.001	< 0.001	< 0.001
**NMN (μmol/day)**	1.89^**¶¶^ (1.20–3.87)	0.19^**^ (0.16–0.22)	0.19^¶¶^ (0.16–0.30)	< 0.001	< 0.001	< 0.001
**Plasma ACTH (pg/mL)**	29.8 (22.8–40.3)	9.7 (8.9–14.1)	20.8 (13.1–38.8)	< 0.001	< 0.001	0.299
**Serum F (μg/dL)**	15.1 (12.3–16.9)	13.6 (12.2–16.4)	15.4 (12.7–17.0)	0.741	–	–
**DHEA-S (μg/dL)**	880 (559–1104)	371 (202–937)	840 (339–1191)	0.562	–	–
**F after 1 mg DST (μg/dL)**	1.5 (1.2–1.7)	6.1 (4.0–10.6)	1.4 (1.3–2.3)	< 0.001	< 0.001	1.000
**Tumor size (cm)**	4.0^¶¶^ (2.5–7.8)	2.7 (2.2–3.4)	2.4^¶¶^ (1.5–3.0)	0.001	0.204	0.001
**CT value (HU)**	36^**¶¶^ (27–42)	15^**^ (6–18)	19^¶¶^ (4–27)	< 0.001	< 0.001	0.003

Data are expressed as median (25-75th percentiles)

*p <0.05 compared between group of PCC and SCS

**p <0.005 compared between group of PCC and SCS

^¶^*p* <0.05 compared between group of PCC and NFA

^¶¶^*p* <0.005 compared between group of PCC and NFA

### PRA, PAC, and ARR in PCC

**Fig. 1:**
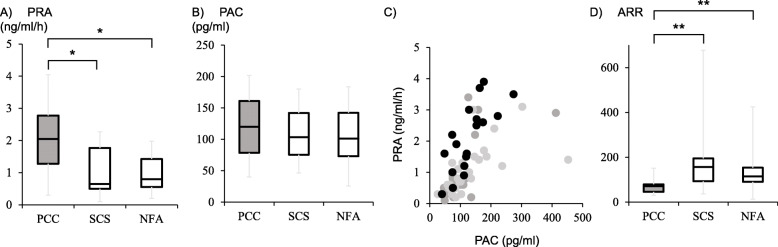
Comparison of plasma renin activity; PRA (**a**), plasma aldosterone concentration; PAC (**b**), Scatter plot of PRA and PAC (**c**), and aldosterone renin ratio; ARR (**d**) in patients with PCC, SCS, and NFA. ARR values were significantly lower in PCC than those in SCS and NFA. Horizontal line, Median; box, 95% CI. *P* values are for the comparisons between all groups by Kruskal-Wallis, followed by Bonferroni’s multiple comparison test between each of the two groups (*p < 0.05, **p < 0.01). Black circle; PCC, dark gray circle; SCS, and light gray circle; NFA

Next, PRA, PAC, and ARR in each group were compared (Fig. 1). PRA was significantly higher in patients with PCC than those with SCS and NFA (2.1 (1.3 ~ 2.8) vs. 0.7 (0.5 ~ 1.8) and 0.9 (0.6 ~ 1.4) ng/mL/h, *p* = 0.018 and *p* = 0.025; Fig. 1a). No difference in PAC was noted among the three groups (Fig. 1b). When PAC and PRA were plotted on the graph, the dots of PCC tended to present on the upper left part, suggesting that PRA was relatively higher than PAC (Fig. 1c). Intriguingly, ARR were significantly lower in patients with PCC than those in patients with SCS and NFA (70.5 (45.5 ~ 79.5) vs. 156.0 (92.9 ~ 194.5) and 114.9 (90.1 ~ 153.4), *p* < 0.001 and *p* < 0.001; Fig. 1d). Clinically, the diagnosis of PCC in patients showing either MN or NMN levels within two-fold of upper limit is challenge. Therefore, we investigated these subjects in our patients (MN; *n* = 7, NMN; *n* = 4, Both; *n* = 1, total; *n* = 10). In these subjects, lower ARR was shown compared to SCS and NFA (PCC vs. SCS and NFA, 72.6 (57.9 ~ 79.5) vs. 156.0 (92.9 ~ 194.5) and 114.9 (90.1 ~ 153.4), *p* = 0.008, respectively), demonstrating the additive utility of ARR in PCC diagnosis.

**Fig. 2:**
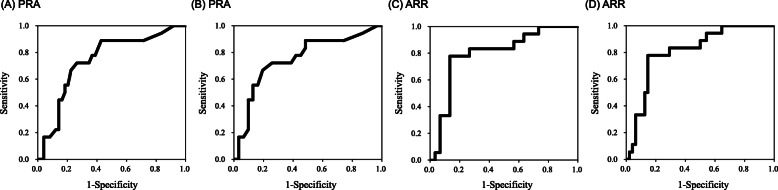
**a** ROC curves of PRA for PCC from SCS and NFA. **b** ROC curve of PRA for PCC from NFA. **c** ROC curves of ARR for PCC from SCS and NFA. **d** ROC curve of ARR for PCC from NFA

ROC curve analysis to determine the optimal cutoff value in the diagnosis of PCC revealed that PRA of 1.45 ng/mL/h was optimal in the differentiation of PCC from SCS and NFA, with a sensitivity of 72.2% and specificity of 73.5% (Table 3a, Fig. 2a). ARR of 80.9 represented the optimal cutoff value with a sensitivity of 77.8% and specificity of 85.4% (Table 3a, Fig. 2c). ARR of 95.4 represented the optimal cutoff value for the differentiation of PCC from NFA, with a sensitivity of 83.3% and specificity of 86.7% (Table 3b, Fig. 2d), while uNMN or the sum of uMN and uNMN level showed a sensitivity of 100, 78% and specificity of 75, 93%, respectively. To test whether ARR could be an additional marker to differentiate PCC from SCS and NFA, AUC (95%CI) for ROC analysis was calculated, revealing that the AUC with uMN, uNMN, and ARR 0.944 (0.889–0.999) was higher than that with uMN and uNMN 0.872 (0.766–0.978), indicating the clinical utility of ARR in PCC diagnosis.

**Table 3:** Receiver Operating Characteristic (ROC) curves

	cutoff value	AUC	Sensitivity	Specificity	***p value***	confidence interval
(A) ROC curves for PCC from SCS and NFA.						
**PRA**	1.45	0.741	72.2	73.5	0.003	0.609–0.874
**ARR**	80.9	0.811	77.8	85.4	< 0.001	0.700–0.923
**uMN + uNMN**	0.146	0.872	77.8	92.6	< 0.001	0.766–0.978
**PRA + uMN + uNMN**	0.132	0.935	88.9	82.6	< 0.001	0.876–0.993
**ARR + uMN + uNMN**	0.213	0.944	94.4	86.7	< 0.001	0.889–0.999
(B) ROC curves for PCC from NFA.						
**PRA**	1.55	0.751	70.0	80.6	0.004	0.603–0.899
**ARR**	95.4	0.858	83.3	86.7	< 0.001	0.764–0.983
**uMN + uNMN**	0.218	0.859	61.1	100	< 0.001	0.743–0.975
**PRA + uMN + uNMN**	0.819	0.946	94.4	83.3	< 0.001	0.890–1.003
**ARR + uMN + uNMN**	0.718	0.939	94.4	86.2	< 0.001	0.872–1.006

*PRA* plasma renin activity, *ARR* aldosterone renin ratio

(A) uMN + uNMN; −2.738 + 8.019 × uMN −0.002 × uNMN, PRA + uMN + uNMN; −3.314 + 0.319 × PRA + 8.111 × uMN - 0.002 × uNMN, ARR + uMN + uNMN; − 0.054 -0.028 × ARR + 10.076 × uMN - 0.004 × uNMN

(B) uMN + uNMN; 2.203–6.983 × uMN + 0.003 × uNMN, PRA + uMN + uNMN; 2.798–0.338 × PRA - 7.032 × uMN - 0.003 × uNMN, ARR + uMN + uNMN; − 0.584 + 0.031 × ARR - 9.848 × uMN + 0.005 × uNMN

## Discussion

In this study, we demonstrated that patients with PCC exhibited low ARR compared with those with SCS or NFA. ARR provided substantial sensitivity and specificity to discriminate PCC from other AIs. Increased ARR has generally been used in the screening of primary aldosteronism in patients with hypertension or AIs. Therefore, both PAC and PRA are commonly measured in the routine diagnostic process in AIs and suspicion of secondary hypertension. The present data suggest that the application of ARR in the screening of PCC as in primary aldosteronism may be useful.

Measurement of plasma MN and uMN levels in the diagnosis of PCC shows excellent sensitivity (70.8–100.0% and 80.0–97.0%) and specificity (79.4–97.6% and 69.0–95.1%) [3–7]; therefore, it has been used as a gold standard. Indeed, in the present study, increased levels of uMN and uNMN provided the highest sensitivity (80 and 100%, respectively) and specificity (100 and 75%, respectively) in the diagnosis of PCCs with an area under the ROC curve of 0.875 and 0.950, respectively. Thus, our data supports that measurements of MN level are recommended to screen PCC as demonstrated in the guidelines [11].

However, in clinical practice, there are some atypical AIs, such as PCCs without an increase in MN level and NFAs with a marginal increase in MN level [14]. In our series, we encountered a patient with NFA who showed increased plasma NA and uNMN levels (plasma NA, 0.63 pmol/mL [reference range, 0.15–0.57 pmol/mL], uNMN, 0.186 μg/day [reference range, 0.029–0.120 μg/day]). Additionally, the patient had bilateral adrenal tumors (2.8 cm on the left, 1.9 cm on the right). ^123^I-MIBG scintigraphy demonstrated that accumulation was detected only in the left tumor. This patient underwent left adrenalectomy, and the final diagnosis was NFA. Preoperative endocrinological examination showed PRA of 0.2 ng/mL/h, PAC of 85 pg/mL, and ARR of 425. To exclude primary aldosteronism, we repeated the examination showed low PRA (0.3 ng/mL/hr), with rather less PAC (31.9 pg/mL), suggesting this high ARR might be due to pseudo-aldosteronism rather than autonomous aldosterone hypersecretion. In fact, both PRA and PAC were not altered after adrenalectomy (0.6 ng/ml/hr. and 57 pg/mL, respectively). These data suggest that the evaluation of ARR provided additional information for accurate diagnosis. Furthermore, PCCs with normal MN levels have also been reported [8, 15]. In such cases, ARR may be helpful in the diagnosis of PCC as an additional marker. In fact, our data suggested ARR was useful in patients with PCC, who showed MN level within two-fold of upper limit.

**Fig. 3:**
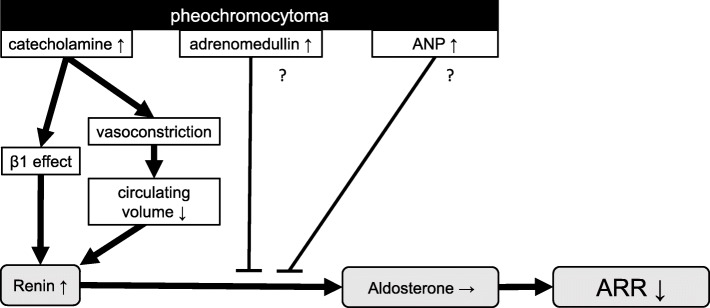
The scheme of mechanistic hypothesis of the decreased ARR in PCC. Despite of the increased renin activity, patients with PCC showed a relatively low aldosterone levels than those in patients with SCS or NFA. This relatively low aldosterone in patients with PCC may be because of the increased secretion of adrenomedullin and ANP

It is well known that increased CA secretion in PCC causes chronic vasoconstriction, resulting in low circulating volume, which increases PRA [9]. Moreover, CAs directly stimulate renin secretion via a β1-adrenergic receptor-mediated process [10] (Fig. 3), leading to increased PRA in PCCs (Fig. 1a). In contrast, there were no differences in PAC between the three groups (Fig. 1b). Interestingly, ROC curve analysis revealed that low ARR had higher sensitivity and specificity than increased PRA to discriminate PCC. These data suggest that aldosterone levels were relatively low despite the increased PRA in each case of PCC. Indeed, when PAC and PRA were plotted on the graph, the dots of PCC tended to present on the upper left part, suggesting that PAC was relatively lower than PRA in each case (Fig. 1c). There are several plausible explanations for this phenomenon. First, adrenomedullin (AM) is a peptide hormone that lowers blood pressure via vasodilation, which was originally isolated from PCC, and has been shown to induce renin resistance with ARR suppression [16]. Since plasma AM level is generally high in PCC [17], this could suppress ARR. Another possibility is atrial natriuretic polypeptide (ANP), whose plasma levels are generally increased in PCC [18]. ANP reduces angiotensin II-dependent aldosterone secretion that causes decreased ARR. Moreover, ANP-dependent renal sodium excretion also leads to suppression of aldosterone production [18–20] (Fig. 3).

Regarding preoperative preparation of PCC, it is critical to normalize circulating volume by α-blocker administration to prevent perioperative complications [21]. In this aspect, it is important to have multiple biomarkers in the diagnosis or exclusion of PCC in addition to CAs preoperatively. Computed tomography (CT) value < 10 HU is a useful marker to rule out PCC in AIs [22]. However, CT value is high in various pathological conditions, such as adrenocortical carcinoma and metastatic adrenal mass. ^123^I-MIBG scintigraphy is also quite useful in the diagnosis of PCC with sensitivity of 85–88% and specificity of 84–100% [23–26]. However, the availability of this imaging equipment is limited especially in primary physicians. It is considered that the additional use of the convenient biomarker ARR in combination with the general information may help in obtaining a more accurate diagnosis.

There are several limitations in this study. This is a retrospective study, and the sample size is small, so it is necessary to validate these results in a large-scale cohort study. In addition, because there were few borderline MN and NMN values patients in this study, we cannot conclude whether ARR was really useful for these borderline cases. Further larger scale investigation is necessary to verify this point. Additionally, because some patients with NFA did not undergo surgery, we cannot completely exclude the possibility that PCC may be present in the NFA group. In this study, the patients taking α-blocker was included. Since α-blocker can restore the circulating plasma volume in patients with PCC, the effect of this drug on lowering PRA levels cannot be excluded. In this study, PCC patients taking α-blocker included 3 subjects. These indicate that ARR can be used as an additional marker even if these drugs have been taken. Finally, because postoperative ARR could not be sufficiently studied, further longitudinal prolong evaluation is required to clarify whether this convenient index could be a useful biomarker at the post-operative state. To simplify the design, we restricted the patients in this study to α-blocker and/or CCB users only. However, in the primary care clinic, it sometimes difficult to adjust the drugs for screening test. We think further investigation will be required whether this convenient ARR will also be useful in patients using other antihypertensive drugs such as β-blocker, diuretics, and ARB/ACEi. β-blockers are expected to increase ARR and diuretics and ARB/ACEi decrease [27]. However, the antihypertensive drugs might be adjusted for screening of adrenal-dependent hypertension including PA in patients with adrenal incidentalomas. We think this study could be helpful at least in these groups.

## Conclusion

We demonstrated that patients with PCC exhibited low PAC/PRA than those with NFA or SCS, indicating that low ARR indicated the possibility of PCC. Further investigation is necessary to clarify whether this convenient index, “ARR,” will help in the diagnosis of PCC.

## Data Availability

The datasets used and/or analysed during the current study are available from the corresponding author on reasonable request.

## Abbreviations

PRA: Plasma renin activity
PCC: Pheochromocytoma
ARR: Aldosterone renin ratio
PAC: Plasma aldosterone concentration
SCS: Subclinical Cushing’s syndrome
NFA: Non-functioning adenoma
ROC: RECEIVER Operator Characteristic
CA: Catecholamine
Ad: Adrenaline
NA: Noradrenaline
DA: Dopamine
MN: Metanephrine
NMN: Normetanephrine
AI: Adrenal incidentaloma
ACE: Angiotensin converting enzyme
ARB: Angiotensin receptor blocker
MIBG: Metaiodobenzylguanidine
HPLC: High performance liquid chromatography
EIA: Enzyme immunoassay
RIA: Radioimmunoassay
BMI: Body mass index
CCB: Calcium channel blocker
ACTH: Adrenocorticotropin
F: Cortisol
DST: Dexamethasone suppression test
DHEA-S: Dehydroepiandrosterone sulfate
CT: Computed tomography
AM: Adrenomedullin
ANP: Atrial natriuretic polypeptide
